# Lymph node ratio is associated with adverse clinicopathological features and is a crucial nodal parameter for oral and oropharyngeal cancer

**DOI:** 10.1038/s41598-017-07134-7

**Published:** 2017-07-27

**Authors:** Zhien Feng, Qiao Shi Xu, Chong Wang, Jin Zhong Li, Ming Hui Mao, Hua Li, Li Zheng Qin, Zhengxue Han

**Affiliations:** Department of Oral and Maxillofacial-Head and Neck Oncology, Beijing Stomatological Hospital, Capital Medical University, Beijing, 100050 P.R. China

## Abstract

The lymph node ratio(LNR) has been described as a novel predictor of the survival of patients with oral and oropharyngeal squamous cell carcinoma(O/OPSCC). The purpose of this study was to evaluate whether LNR is better at predicting survival and the need for adjuvant treatment than traditional tumour-nodal-metastasis(TNM) staging. Eight hundred nine patients with O/OPSCC and positive lymph node disease were retrospectively enrolled in this study. LNR equal to 0.075 is the best cut-off value for stratifying 5-year disease-free survival(DFS). High LNR is closely associated with more advanced T stage, higher N stage, more severe pathological grade, the presence of diffuse infiltration and extracapsular spread(ECS). LNR is better for evaluating prognosis than the pathological N stage. Patients with high LNR coupled with high number of positive lymph nodes who received adjuvant concurrent chemo-radiotherapy(CCRT) had a better 5-year DFS than patients who received surgery alone. Multivariate analyses revealed that T stage, ECS and LNR are independent prognostic factors of 5-year DFS and disease-specific survival(DSS). Therefore, high LNR is closely correlated with adverse parameters that markedly hinder prognosis. LNR is superior to traditional TNM staging for the evaluation of prognosis,and the combination of the LNR with the number of positive lymph nodes can predict the benefits of adjuvant CCRT.

## Introduction

The lymph node status has been recognized as one of the most significant prognostic factors for patients with oral and oropharyngeal squamous cell carcinoma (O/OPSCC)^1–4^. In clinical practice, the presence of a metastatic lymph node markedly reduces the potential for a favourable prognosis^5^. Currently, only the American Joint Committee on Cancer (AJCC/UICC) Staging System, which stages the neck based on the following nodal parameters: number, size and site of positive lymph nodes, is used^6^. Based on accumulating evidence, factors such as the presence of extracapsular spread (ECS), the number of positive nodes, the lymph node ratio (LNR) and even the lymph node yield (LNY) are important prognostic factors that have not been incorporated in staging systems^7^. Therefore, the development of better risk stratification based on these nodal parameters for patients with O/OPSCC is a crucial need^8^.

The LNR, which is defined as the ratio of the number of positive lymph nodes to the total number of excised lymph nodes, was recently introduced as an important diagnostic tool for O/OPSCC^9^. In recently published studies, the LNR was used as an independent prognostic factor for O/OPSCC and was used to predict the benefits of adjuvant radiotherapy (RT)/chemo-radiotherapy (CCRT)^9, 10^. However, other studies reported controversial findings. For example, because the extent of neck dissection is clearly associated with the number of harvested lymph nodes, the LNR appears to strongly depend on the harvesting protocol, specimen processing procedures and the extent of the neck dissection^11, 12^. Therefore, the predictive value of the LNR for the prognosis and treatment of HNCSS and its advantages compared with traditional tumour-nodal-metastasis (TNM) staging remain uncertain.

Although tumour staging according to the traditional TNM system has been widely accepted, treatment management and prognostic judgement based on these classifications alone remain insufficient because TNM staging is based on the simplistic concept of an orderly progression of the primary tumour located in surrounding tissues to lymphatic and vascular metastasis^9^. Due to the complex heterogeneity of O/OPSCC diseases, many other prognostic parameters should be investigated to acquire the best clinical evidence and to meet the demand for personalized diagnosis and treatment^13^.

Based on the results from our previous studies, the LNY alone is not a better predictive factor for long-term survival^14, 15^. In addition, the prognostic value of other nodal parameters, such as the LNR, ECS and the number of positive nodes, in a northern Chinese population compared with the TNM stage remains unclear. The purpose of this study was to explore whether the LNR is a more valid predictive factor for treatment choice, tumour relapse and long-term survival than the TNM staging system and other parameters, including ECS and the number of positive nodes. In addition, if the LNR is a better predictive factor, we also sought to determine the best LNR cut-off value for classifying patients with nodal positive (pN+) O/OPSCC into high- or low-risk populations to aid surgeons in adopting personalized diagnoses and treatment strategies.

## Results

### Patients and baseline data

Eight hundred nine patients with O/OPSCC were consecutively enrolled in the study, including 546 (67.5%) men and 263 (32.5%) women. The median age was 58.0 years (range: 16–89 years). Most patients had a tumour in the tongue (37.3%), followed by the lower gingiva (15.8%), buccal tissue (13.6%), floor of the mouth (11.0%), oropharynx (14.0%), upper gingiva (6.6%), and hard palate (1.7%). The T staging was: T1 (n = 80, 9.9%), T2 (n = 306, 37.8%), T3 (n = 95, 11.7%), T4a (n = 301, 37.2%), and T4b (n = 27, 3.4%). Regarding the pathological grade, 201 patients (24.8%) had grade I tumours, 526 (65.0%) had grade II tumours, 75 (9.3%) had grade III tumours, and 7 (0.9%) had missing data. Three hundred forty-six successive cases were available for the analysis of histological signs of severity (perineural invasion, vascular emboli, and diffuse infiltration). Specifically, perineural invasion was present in 91 cases and absent in 255 cases. Vascular emboli were present in 22 cases and absent in 324 cases. Diffuse infiltration was present in 142 cases and absent in 204 cases. All margins of the resected tumours were negative, according to the pathological reports (Table 1).

**Table 1:** Baseline demographics of the 809 patients included in this study.

Variable	No.	%	Low LNR (n = 386)		High LNR (n = 423)		*P*
			No.	%	No.	%	
**Age, yrs: mean ± SD**	57.0 ± 11.9	56.4 ± 11.7	57.6 ± 11.9	0.155			
**Gender**							
Male	546	67.5	256	66.3	290	68.6	0.498
Female	263	32.5	130	33.7	133	31.4	
**Sites**							
Tongue	302	37.3	143	37.0	159	37.6	0.063
Lower gingiva	128	15.8	59	15.3	69	16.3	
Buccal mucosa	110	13.6	48	12.4	62	14.7	
Floor of the mouth	89	11.0	37	9.6	52	12.3	
Oropharynx	113	14.0	70	18.1	43	10.2	
Upper gingiva	53	6.6	23	6.0	30	7.1	
Hard palate	14	1.7	6	1.6	8	1.9	
**Oral cavity and oropharynx**							
Oral cavity	696	86.0	316	81.9	380	89.8	0.001
Oropharynx	113	14.0	70	18.1	43	10.2	
**T stage**							
T1	80	9.9	56	14.5	24	5.7	<0.001
T2	306	37.8	165	42.7	141	33.3	
T3	95	11.7	41	10.6	54	12.8	
T4a	301	37.2	110	28.5	191	45.2	
T4b	27	3.4	14	3.6	13	3.1	
**N stage**							
N1	340	42.0	296	76.7	44	10.4	<0.001
N2b	410	50.7	85	22.0	325	76.8	
N2c	59	7.3	5	1.3	54	12.8	
**Pathological grade**							
I	201	24.8	116	30.1	85	20.1	0.005
II	526	65.0	235	60.9	291	68.8	
III	75	9.3	32	8.3	43	10.2	
Missing	7	0.9	3	0.8	4	0.9	
**Growth pattern**							
Exophytic	170	21.0	90	23.3	80	18.9	0.381
Ulcerative	204	25.2	98	25.4	106	25.1	
Infiltrative	357	44.1	166	43.0	191	45.2	
Missing	78	9.6	32	8.3	46	10.9	
**Smoking history**							
Smoker	436	53.9	199	51.6	237	56.0	0.251
Non-smoker	360	44.5	179	46.4	181	42.8	
Missing	13	1.6	8	2.0	5	1.2	
**Alcohol history**							
Drinker	350	43.3	166	43.0	184	43.5	0.976
Non-drinker	446	55.1	212	54.9	234	55.3	
Missing	13	1.6	8	2.1	5	1.2	
**Perineural invasion**							
Absence	255	73.7	138	76.2	117	70.9	0.260
Presence	91	26.3	43	23.8	48	29.1	
**Vascular emboli**							
Absence	524	93.6	173	95.6	151	91.5	0.122
Presence	22	6.4	8	4.4	14	8.5	
**Diffuse infiltration**							
Absence	204	59.0	119	65.7	85	51.5	0.007
Presence	142	41.0	62	34.3	80	48.5	
**Extracapsular spread**							
Absence	230	60.2	139	72.4	91	47.9	<0.001
Presence	152	39.8	53	27.6	99	52.1	
**Neck dissection**							
SND	357	44.1	173	44.8	184	43.5	0.706
CND	452	55.9	213	55.2	239	56.5	
**Management**							
Surgery alone	111	13.7	62	16.1	49	11.6	<0.001
Surgery + RT	540	66.7	251	65.0	289	68.3	
Surgery + CCRT	114	14.2	30	7.8	84	19.9	
Missing	44	5.4	43	11.1	1	0.2	

Notes: LNR, lymph node ratio; SD, standard deviation; SND, selective neck dissection; CND, comprehensive neck dissection; RT, radiotherapy; CCRT, concurrent chemo-radiotherapy.

The 809 patients underwent 981 lateral neck dissections, and 172 patients underwent bilateral neck dissection. The largest proportion of patients was treated with comprehensive neck dissection (n = 452, 55.9%), followed by selective neck dissection (n = 357, 44.1%). The lymph node status of 50.7% of patients was pN2b, followed by 42.0% of patients with pN1, and 7.3% of patients with pN2c. The mean and median LNY values in each lateral lymph node were 27.3 and 25.0, respectively (range: 10–76). The mean number of positive lymph nodes was 2.8 (range: 1–55). In pN + patients, the majority of cases were single lymph node metastasis (43.4%), followed by two lymph node metastases (21.1%). The mean and range of the LNR were 10% and 1.5–77.4%, respectively. Three hundred eighty-two successive cases were available for the analysis of the extracapsular spread (ECS) status. The ECS status was positive in 152 cases and negative in 230 cases. One hundred eleven (13.7%) patients received surgery alone, 540 (66.7%) patients received adjuvant radiotherapy, 114 (14.2%) patients received concurrent chemo-radiotherapy, and data for 44 (5.4%) patients were missing (Table 1).

### Cut-off values for nodal parameters

**Figure 1:**
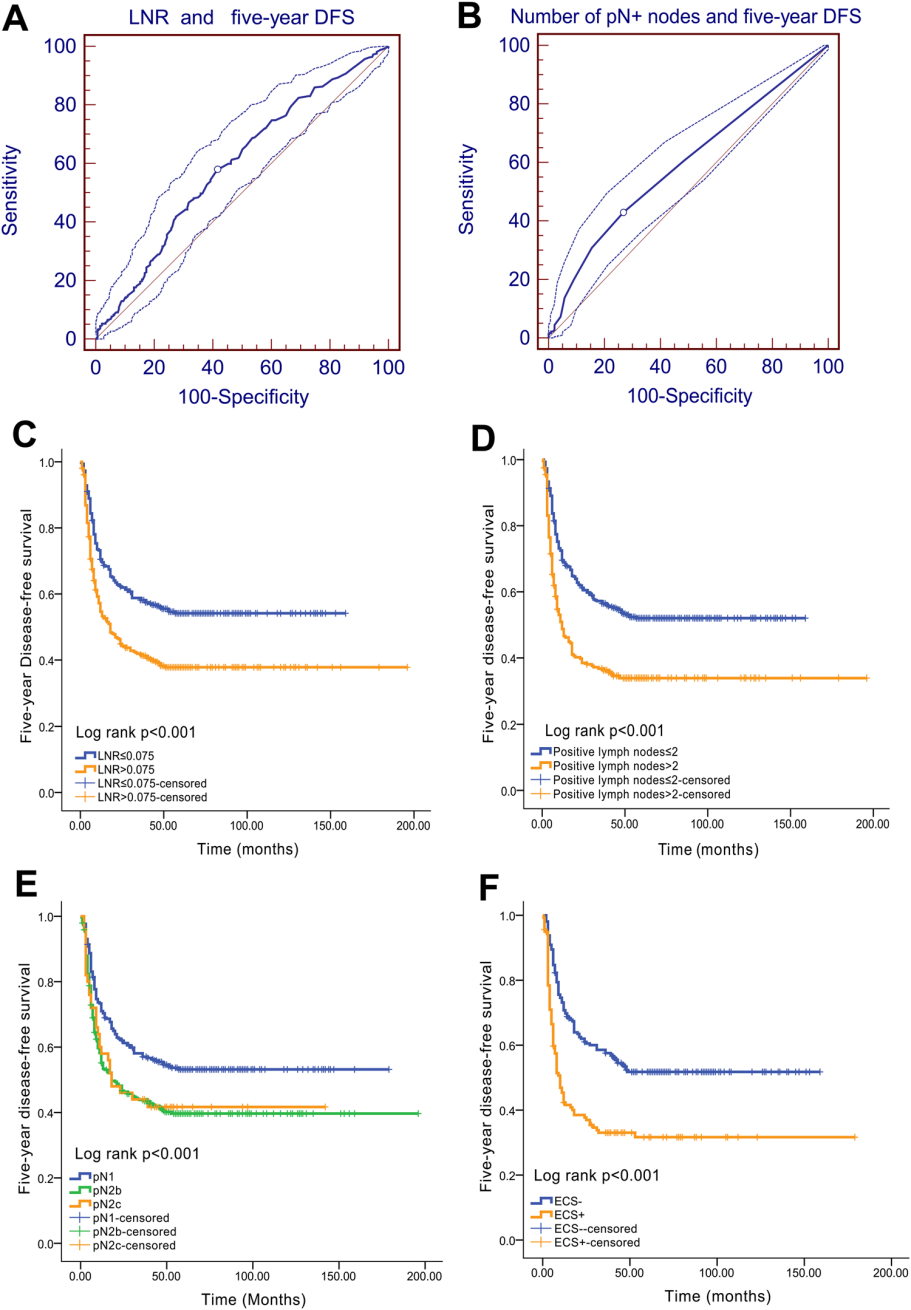
Best cut-off values for nodal parameters and 5-year DFS. (**A**) ROC curve analysis of the LNR cut-off value and 5-year DFS (the cut-off value for the LNR is 0.075); (**B**) ROC curve analysis of the number of positive lymph nodes and 5-year DFS (the cut-off value for the number of positive lymph nodes is 2). (**C**) Five-year DFS curve for the LNR; (**D**) 5-year DFS curve for the number of positive lymph nodes; (**E**) 5-year DFS curve for the pN stage; (**F**) 5-year DFS curve for the ECS status.

**Figure 2:**
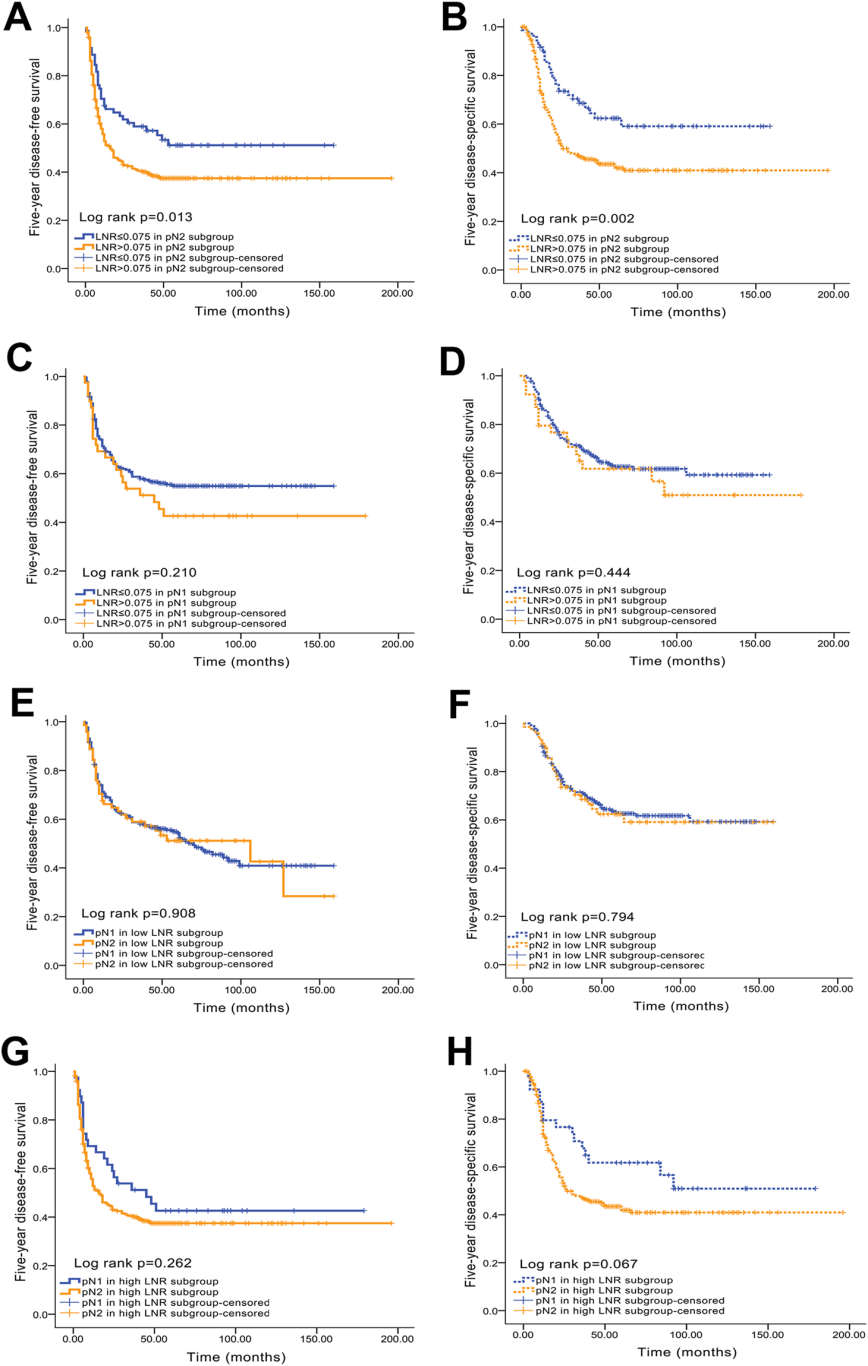
Kaplan-Meier analyses of 5-year DFS and DSS between the LNR and pN stage. (**A**) Five-year DFS curve for the LNR in the pN2 subgroup; (**B**) 5-year DSS curve for the LNR in the pN2 subgroup; (**C**) 5-year DFS curve for the LNR in the pN1 subgroup; (**D**) 5-year DSS curve for the LNR in the pN1 subgroup; (**E**) 5-year DFS curve for pN in the low LNR subgroup; (**F**) 5-year DSS curve for pN in the low LNR subgroup; (**G**) 5-year DFS curve for pN in the high LNR subgroup; (**H**) 5-year DSS curve for pN in the high LNR subgroup.

The patients were divided into high- or low-risk subgroups using the best cut-off values for the nodal parameters. For continuous variables (including the LNR and the number of positive lymph nodes), the cut-off values for predicting 5-year disease-free survival (DFS) were first determined based on an analysis of the receiver operating characteristic (ROC) curves. Subsequently, all patients were allocated into the high or low subgroups according to the cut-off value (the continuous variables were replaced with categorical variables). The differences in 5-year DFS between the high-risk and low-risk subgroups were then analysed using Kaplan-Meier method and log-rank tests, as described by Liao *et al*.^7^. In addition, the cut-off values for categorical variables (including ECS and the traditional N stage) were determined based on the ECS status (ECS^−^ or ECS^+^) and N stage (N1, N2b and N2c). According to the analysis of the ROC curves, the best cut-off values for the LNR and the number of positive lymph nodes were 0.075 (area under the curve (AUC): 0.592, sensitivity: 58.0%, specificity: 58.4%, *P = *0.0001, Fig. 1A) and 2 (AUC: 0.593, sensitivity: 42.9%, specificity: 73.3%, *P = *0.0001, Fig. 1B), respectively. All patients were divided into different subgroups according to their cut-off values. Patients with primary tumours located in the oral cavity have a greater proportion of a high LNR than patients with primary tumours located in the oropharynx subsite (*P* < 0.001, Table 1).

The 5-year DFS differed significantly if patients were stratified according to the LNR (low LNR vs. high LNR: 55.7% vs. 39.8%, *P* < 0.001, Fig. 1C), the number of positive lymph nodes (≤2 vs. >2: 53.7% vs. 36.1%, *P* < 0.001, Fig. 1D), the pN stage (pN1 vs. pN2a vs. pN2b: 54.6% vs. 41.9% vs. 42.0%, *P* < 0.001, Fig. 1E), and the ECS status (ECS^−^ vs. ECS^+^: 52.6% vs. 33.8%, *P* < 0.001, Fig. 1F).

### Subsite analysis

According to the Kaplan-Meier analysis, patients with stage III oral cancer have a similar 5-year disease-specific survival (DSS) compared with patients with oropharyngeal cancer (70.7% vs. 73%, *P* = 0.755). In addition, patients with stage IV oral cancer have a similar 5-year DSS compared with patients with oropharyngeal cancer (50.2% vs. 40.4%, *P* = 0.121). In this study, only 13 cases of all oropharyngeal cancers were analysed for p16^(INK4A)^ expression through immunohistochemistry and human papillomavirus 16/18 (HPV16/18) expression using a specific *in situ* hybridization protocol. Only 2 cases were identified as HPV-positive, and the remaining 11 cases were HPV-negative.

### A high LNR is closely associated with many poor prognostic factors for O/OPSCC

According to the chi-square test, a high LNR was closely associated with a more advanced T stage (*P* < 0.001), a higher N stage (*P* < 0.001), a more severe pathological grade (*P* = 0.005), and the presence of diffuse infiltration (*P* = 0.007) and ECS (*P* < 0.001). Therefore, in this study, patients with O/OPSCC and a high LNR underwent more adjuvant RT or CCRT than those with a low LNR (*P* < 0.001).

### Patterns of failure

During the follow-up period, 351 (43.4%) of the 809 patients survived, 342 (42.3%) died, and 116 (14.3%) were lost to follow-up. Twenty-four patients died due to causes unrelated to cancer, and these included 5 patients who died of cardiac failure and brain stroke, 5 patients who died of multiple organ failure, 8 patients who died of respiratory failure, 1 patient who died of acute gastrointestinal haemorrhage, 1 patient who died of suicide, 1 patient who died of septicaemia, and 3 patients who died of uncertain causes. Cumulatively, 385 of the 809 patients (47.6%) experienced disease relapse. The first relapse was local recurrence alone in 178 (22.0%) patients and regional recurrence alone in 120 (14.8%) patients. Fifty (6.2%) patients developed second primary carcinomas as a first event (which was simultaneously coupled with neck recurrence in 1 patient). Forty (4.9%) patients developed distant metastasis (which was simultaneously coupled with neck recurrence in 2 patients).

### LNR is advantageous in evaluating prognoses compared with the pathological N stage

Both the 5-year DFS and 5-year DSS differed significantly if the patients were stratified according to the LNR. The log-rank test revealed *P* < 0.001 for both DFS and DSS. Five-year DFS and 5-year DSS remained significant when the LNR was used if only patients with a pN2 status were included in the analysis. Among these patients, individuals with an LNR ≤ 0.075 had a significantly more favourable prognosis than patients with an LNR > 0.075, with *P* values of 0.013 for DFS (53.5% vs. 39.4%, Fig. 2A) and 0.002 for DSS (64.8% vs. 45.9%, Fig. 2B). However, pN1 patients with an LNR ≤ 0.075 did not have markedly better prognoses for both 5-year DFS (56.3% vs. 43.6%, *P* = 0.210, Fig. 2C) and 5-year DSS (65.4% vs. 59.0%, *P* = 0.444, Fig. 2D) than patients with an LNR > 0.075.

Conversely, in the low-LNR subgroup (LNR ≤ 0.075), the 5-year DFS (50.6% for pN1 vs. 50.7% for pN2, *P* = 0.908, Fig. 2E) and 5-year DSS (65.4% for pN1 vs. 64.8% for pN2, *P* = 0.794, Fig. 2F) of patients with different N stages (N1 and N2) were not significantly different. Similarly, no significant differences in 5-year DFS (43.6% for pN1 vs. 39.4% for pN2, *P* = 0.262, Fig. 2G) and 5-year DSS (59.0% for pN1 vs. 45.9% for pN2, *P* = 0.067, Fig. 2H) were observed between patients with different N stages (N1 and N2) in the high LNR subgroup.

### The LNR and ECS have similar predictive values for 5-year DFS and DSS

**Figure 3:**
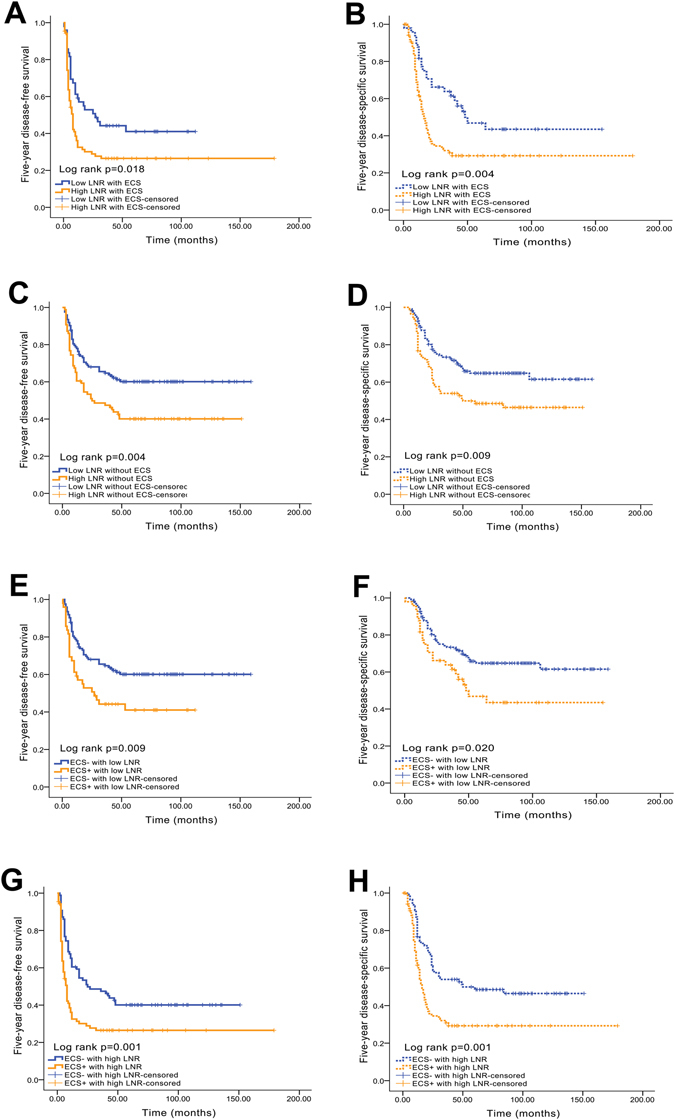
Kaplan-Meier analyses of 5-year DFS and DSS between the LNR and ECS status. (**A**) Five-year DFS curve for the LNR in the ECS^+^ subgroup; (**B**) 5-year DSS curve for the LNR in the ECS^+^ subgroup; (**C**) 5-year DFS curve for the LNR in the ECS^−^ subgroup; (**D**) 5-year DSS curve for the LNR in the ECS^−^ subgroup; (**E**) 5-year DFS curve for the ECS status in the low LNR subgroup; (**F**) 5-year DSS curve for the ECS status in the low LNR subgroup; (**G**) 5-year DFS curve for the ECS status in the high LNR subgroup; (**H**) 5-year DSS curve for the ECS status in the high LNR subgroup.

In patients with ECS, significant differences in prognosis were observed between the high and low LNR groups (5-year DFS for low LNR vs. high LNR: 42.9% vs. 28.7%, *P* = 0.018, Fig. 3A; 5-year DSS for low LNR vs. high LNR: 51.0% vs. 33.3%, *P* = 0.004, Fig. 3B). Similarly, patients without ECS who had a high LNR exhibited poorer 5-year DFS (40.7% vs. 61.0%, *P* = 0.004, Fig. 3C) and 5-year DSS (48.8% vs. 65.9%, *P* = 0.009, Fig. 3D) than patients with low LNR. In contrast, in both the low and high LNR subgroups, significant differences in prognosis were observed when the ECS status was compared (5-year DFS in the low LNR subgroup—ECS^−^ vs. ECS^+^: 61.0% vs. 42.9%, *P* = 0.009, Fig. 3E; 5-year DSS in the low LNR subgroup—ECS^−^ vs. ECS^+^: 65.9% vs. 51.0%, *P* = 0.020, Fig. 3F; 5-year DFS in the high LNR subgroup—ECS^−^ vs. ECS^+^: 40.7% vs. 28.7%, *P* = 0.001, Fig. 3G; 5-year DSS in the high LNR subgroup—ECS^−^ vs. ECS^+^: 48.8% vs. 33.3%, *P* = 0.001, Fig. 3H).

### LNR versus the number of positive lymph nodes

When the number of positive lymph nodes is considered the sole factor, the 5-year DFS (≤2 vs. >2: 53.7% vs. 36.1%, *P* < 0.001) and 5-year DSS (≤2 vs. >2: 64.4% vs. 40.6%, *P* < 0.001) significantly differed. However, if patients with a low LNR (LNR ≤ 0.075) were stratified by the number of positive lymph nodes, the number of positive lymph nodes did not significantly affect the 5-year DFS (55.7% vs. 54.5%, *P* = 0.992) or 5-year DSS (65.3% vs. 63.6%, *P* = 0.796). Similarly, if patients with a low number of positive lymph nodes (≤2) were stratified based on the cut-off value for the LNR, the LNR did not significantly affect the 5-year DFS (55.7% vs. 48.4%, *P* = 0.231) or 5-year DSS (65.3% vs. 61.9%, *P* = 0.342).

### LNR and ECS are better at predicting long-term survival than the traditional TNM stage

No significant difference in the 5-year DSS of patients with a high LNR was observed after the data were stratified by TNM stage (stage III vs. stage IV: 46.7% vs. 39.2%, *P* = 0.219). However, significant differences in the 5-year DFS (low LNR vs. high LNR: 49.3% vs. 39.2%, *P* = 0.015) and 5-year DSS (low LNR vs. high LNR: 56.8% vs. 45.6%, *P* = 0.004) of patients with stage IV disease were observed after the data were stratified based on the cut-off value for the LNR.

Similarly, no significant differences in the 5-year DFS (stage III vs. stage IV: 42.1% vs. 32.5%, *P* = 0.311) or 5-year DSS (stage III vs. stage IV: 42.1% vs. 39.3%, *P* = 0.379) were observed for ECS^+^ patients after the data were stratified by the TNM stage. However, significant differences in the 5-year DFS (ECS^−^ vs. ECS^+^: 62.2% vs. 42.1%, *P* = 0.031) and 5-year DSS (ECS^−^ vs. ECS^+^: 70.0% vs. 42.1%, *P* = 0.005) of patients with stage III disease were observed after the data were stratified based on the cut-off value for the ECS status. Significant differences in the 5-year DFS (ECS^−^ vs. ECS^+^: 45.4% vs. 32.5%, *P* = 0.001) and 5-year DSS (ECS^−^ vs. ECS^+^: 50.4% vs. 39.3%, *P* = 0.003) of patients with stage IV disease were observed after the data were stratified based on the cut-off value for ECS status.

### The number of positive lymph nodes has a similar prognostic value as the traditional TNM stage

Significant differences in the prognosis of patients with lower numbers of positive lymph nodes were observed based on the TNM stage (5-year DFS for stage III vs. stage IV: 58.7% vs. 48.9%, *P* = 0.025; 5-year DSS for stage III vs. stage IV: 71.1% vs. 58.0%, *P* = 0.002). Similarly, patients with stage IV disease and a large number of positive lymph nodes exhibited poorer 5-year DFS (36.1% vs. 48.9%, *P* < 0.001) and 5-year DSS (40.6% vs. 58.0%, *P* < 0.001) than patients with a lower number of positive lymph nodes.

### Patients with both a high LNR and a high number of positive lymph nodes only benefit from adjuvant CCRT

**Figure 4:**
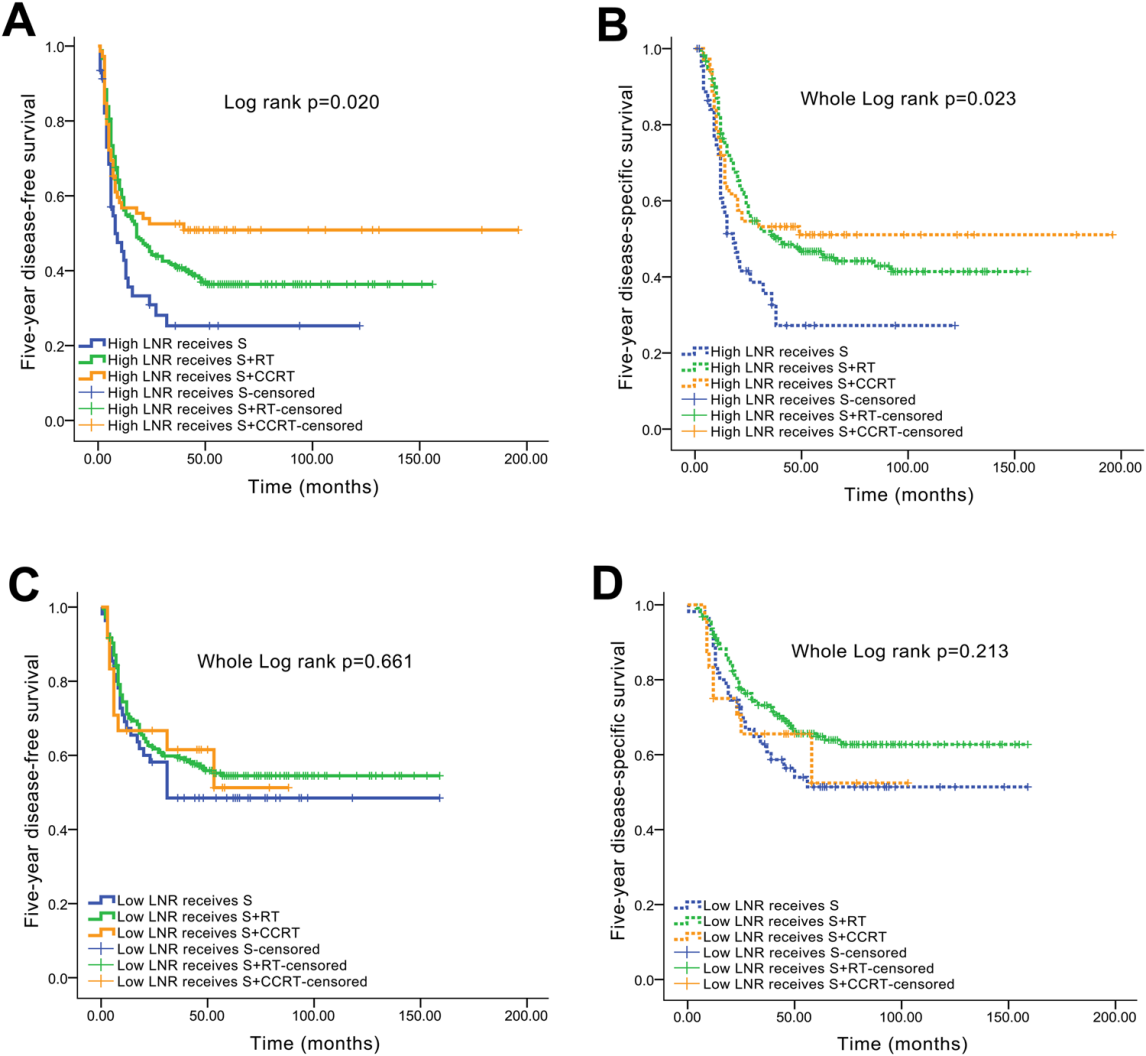
Kaplan-Meier analyses of 5-year DFS and DSS between the LNR and management. (**A**) Five-year DFS curves for patients with a high LNR who received surgery alone, surgery +RT, or surgery +CCRT; (**B**) 5-year DSS curves for patients with a high LNR who received surgery alone, surgery +RT, or surgery +CCRT; (**C**) 5-year DFS curves for patients with a low LNR who received surgery alone, surgery +RT, or surgery +CCRT; ﻿(**D**) 5-year DSS curves for patients with a low LNR who received surgery alone, surgery +RT, or surgery +CCRT.

With the exception of ECS and/or positive margins, other parameters have not yet been clearly identified as predictors of which patients with O/OPSCC need to receive CCRT. In this study, patients with a high LNR alone who only received surgery had a markedly worse 5-year DFS (30.4%) and 5-year DSS (37.0%) than patients who received surgery and RT (DFS: 38.2%; DSS: 47.7%) or surgery and CCRT (DFS: 51.4%; DSS: 52.8%) (log-rank test for 5-year DFS, *P* = 0.020; log-rank test for 5-year DSS, *P* = 0.023; Fig. 4A and B). However, patients with a low LNR who underwent surgery alone exhibited similar 5-year DFS (49.1%) and 5-year DSS (54.5%) compared with patients who received surgery and RT (DFS: 56.2%; DSS: 67.7%) or surgery and CCRT (DFS: 58.3%; DSS: 62.5%) (log-rank test for 5-year DFS, *P* = 0.661; log-rank test for 5-year DSS, *P* = 0.213; Fig. 4C and D).

**Figure 5:**
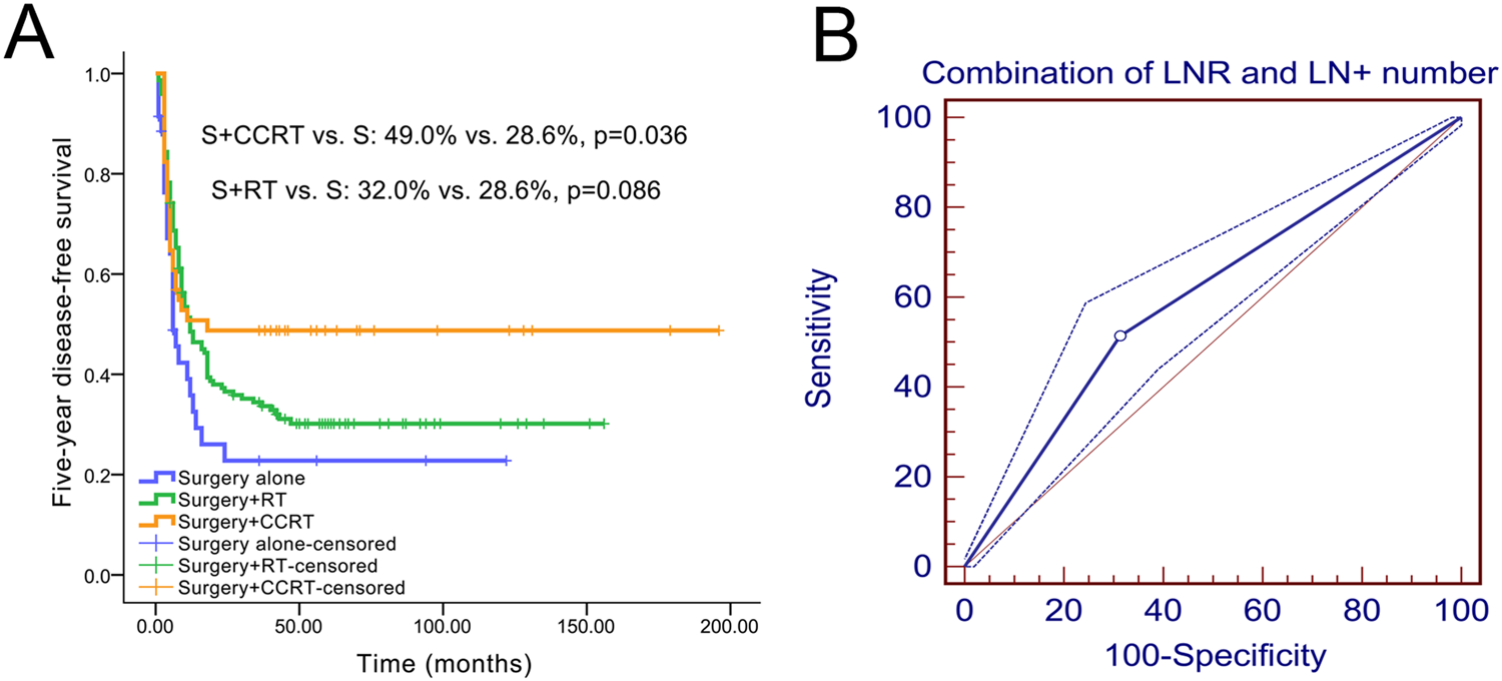
Kaplan-Meier and ROC curve analyses of 5-year DFS between the combination of the LNR and number of positive lymph nodes and management. (**A**) Five-year DFS survival curves; (**B**) 5-year ROC curve.

Interestingly, patients with both a high LNR and a high number of positive lymph nodes (LNR > 0.075 and number of positive lymph nodes > 2) who received surgery and CCRT had a markedly better 5-year DFS than patients who received surgery alone (49.0% vs. 28.6%, *P* = 0.036). In comparison, patients who received surgery and RT had a similar 5-year DFS to patients who received surgery alone (32.0% vs. 28.6%, *P* = 0.086; Fig. 5A). Moreover, based on a further analysis of the ROC curves, the combination of the LNR and the number of positive lymph nodes has a better prognostic value for 5-year DFS than the LNR alone (AUC: 0.600, sensitivity: 51.4%, specificity: 68.7%, *P = *0.0001, Fig. 5B).

### Cox multivariate regression analysis

According to the results of the univariate analyses, the T stage (hazard ratio (HR): 1.234, 95% confidence interval (CI): 1.125–1.353, *P* < 0.001), pN stage (HR: 1.331, 95% CI: 1.133–1.563, *P* < 0.001), pathological grade (HR: 1.238, 95% CI: 1.031–1.486, *P* = 0.022), ECS (HR: 1.990, 95% CI: 1.493–2.651, *P* < 0.001), the number of positive lymph nodes (HR: 1.809, 95% CI: 1.469–2.228, *P* < 0.001), and the LNR (HR: 1.639, 95% CI: 1.329–2.021, *P* < 0.001) were closely correlated with 5-year DFS. All parameters included in the univariate analysis were further assessed using a Cox multivariate regression analysis (forward method). Based on the results of this analysis, the T stage (HR: 1.204, 95% CI: 1.050–1.381, *P* = 0.008), ECS (HR: 1.754, 95% CI: 1.307–2.353, *P* < 0.001) and LNR (HR: 1.644, 95% CI: 1.220–2.216, *P* = 0.001) were independent predictive factors for 5-year DFS (Table 2).

**Table 2:** Cox proportional hazards regression models estimating 5-year DFS and 5-year DSS.

Variable	HR	95% CI	*P*
**Five-year DFS**			
**Univariate analysis**			
T stage (T1, T2, T3, or T4)	1.234	1.125–1.353	<0.001
pN stage (N1, N2b, or N2c)	1.331	1.133–1.563	<0.001
Pathological grade (I, II, or III)	1.238	1.031–1.486	0.022
ECS (absence vs. presence)	1.990	1.493–2.651	<0.001
Number of positive lymph nodes (≤2 vs. >2)	1.809	1.469–2.228	<0.001
LNR (≤0.075 vs. >0.075)	1.639	1.329–2.021	<0.001
**Multivariate survival analysis (forward method)**			
T stage (T1, T2, T3, or T4)	1.204	1.050–1.381	0.008
ECS (absence vs. presence)	1.754	1.307–2.353	<0.001
LNR (≤0.075 vs. >0.075)	1.644	1.220–2.216	0.001
**Five-year DSS**			
**Univariate analysis**			
Growth pattern (exophytic, ulcerative or infiltrative)	1.244	1.067–1.450	0.005
T stage (T1, T2, T3, or T4)	1.358	1.227–1.503	<0.001
pN stage (N1, N2b, or N2c)	1.525	1.281–1.815	<0.001
Pathological grade (I, II, or III)	1.307	1.073–1.593	0.008
ECS (absence vs. presence)	2.111	1.556–2.863	<0.001
Number of positive lymph nodes (≤2 vs. >2)	2.274	1.815–2.850	<0.001
LNR (≤0.075 vs. >0.075)	1.907	1.513–2.405	<0.001
**Multivariate survival analysis (forward method)**			
T stage (T1, T2, T3, or T4)	1.263	1.066–1.497	0.007
ECS (absence vs. presence)	1.934	1.321–2.830	0.001
LNR (≤0.075 vs. >0.075)	1.740	1.206–2.511	0.003

Notes: LNR, lymph node ratio; pN stage, pathological node stage; ECS, extracapsular spread; RT, radiotherapy; CCRT, concurrent chemo-radiotherapy.

For the analysis of independent predictive factors for 5-year DSS, the first step of the univariate analyses showed that the growth pattern (HR: 1.244, 95% CI: 1.067–1.450, *P* = 0.005), T stage (HR: 1.358, 95% CI: 1.227–1.503, *P* < 0.001), pN stage (HR: 1.525, 95% CI: 1.281–1.815, *P* < 0.001), pathological grade (HR: 1.307, 95% CI: 1.073–1.593, *P* = 0.008), ECS (HR: 2.111, 95% CI: 1.556–2.863, *P* < 0.001), number of positive lymph nodes (HR: 2.274, 95% CI: 1.815–2.850, *P* < 0.001), and LNR (HR: 1.907, 95% CI: 1.513–2.405, *P* < 0.001) were closely correlated with 5-year DSS. All parameters included in the univariate analysis were further analysed through Cox multivariate regression (forward method). Similarly, the T stage (HR: 1.263, 95% CI: 1.066–1.497, *P* = 0.007), ECS (HR: 1.934, 95% CI: 1.321–2.830, *P* = 0.001) and LNR (HR: 1.740, 95% CI: 1.206–2.511, *P* = 0.003) were independent predictive factors for 5-year DSS in this analysis (Table 2).

## Discussion

Although lymph node metastasis is the most important prognostic factor for O/OPSCC, the precise risk stratification of pN + patients using traditional TNM stages is inadequate^16^. Based on the results from our previous studies, the traditional TNM stage might not be the best prognostic factor in the presence of ECS, adjuvant treatments and even some important biomarkers^17, 18^. The LNR has recently been described as a potential predictor of survival and the need for adjuvant treatment in patients with O/OPSCC. However, its predictive value compared with ECS, the number of positive lymph nodes and N stage requires further testing^10, 12^. In this study, we first determined the best cut-off value for the LNR. In addition, we then compared the values among different nodal parameters, including LNR, N stage, ECS and the number of positive lymph nodes, in predicting DFS, DSS and treatment choice.

Based on our results, an LNR equal to 0.075 was the best cut-off value for dividing pN + patients with O/OPSCC into low- and high-risk subgroups according to the 5-year DFS. The use of an LNR of 0.075 as the cut-off value was similar to the results reported by Ebrahimi *et al*.^19^ and Patel *et al*.^20^. In this study, we did not observe a significant difference in the LNR between patients who received selective neck dissection (SND) and comprehensive neck dissection (CND). Therefore, bias from different surgical neck dissection methods was excluded.

A high LNR was closely associated with many adverse clinicopathological factors, including advanced T and N stages, a severe pathological grade, and the presence of diffuse infiltration and ECS. These adverse factors have been widely accepted as negative prognostic parameters and poor pathological characteristics. Although the predictive value of the LNR has never been considered when scheduling treatment strategies, most patients with O/OPSCC and a high LNR underwent more adjuvant RT or CCRT after the primary tumours were resected. Unfortunately, patients with a high LNR still exhibited very poor 5-year DFS (approximately 14% decrease) and DSS (approximately 20% decrease) compared with patients with a low LNR.

In this study, the traditional N stage did not serve as an independent predictive factor for 5-year DFS and DSS. Moreover, no significant differences in 5-year DFS and DSS were observed between pN2b and pN2c patients. Thus, we were unable to easily perform further risk stratification for patients with positive lymph nodal diseases. Based on the results of the comparison analysis, the LNR and ECS have stronger prognostic value than the traditional N stage, according to Cox proportional hazards regression models. If patients were divided into LNR subgroups, the pN stage (pN1 or pN2) would lose its efficacy to accurately predict patient prognoses. Thus, the LNR might be used as an alternative staging system because it is superior to the TNM pathological nodal staging system for predicting recurrence risk and survival after surgery, corroborating the conclusions reported by Ebrahimi *et al*.^21^.

The importance of the ECS status in determining the prognosis of patients with O/OPSCC has been widely accepted^22^. In this study, the LNR influenced the ECS prognosis (ECS^−^ or ECS) of patients, and conversely, the ECS status was used to classify patients with or without a high LNR. In this study, the LNR was closely associated with the ECS status, and a subsequent multivariate analysis also revealed that both LNR and ECS were independent predictive factors for 5-year DFS and DSS. Therefore, we speculate that the LNR and ECS have similar predictive values in patients with O/OPSCC.

Many published reports have verified that patients with a high LNR could benefit more from adjuvant CCRT than from RT or surgery alone for the treatment of solid tumours^10, 23^. In this study, patients with a high LNR had received more rounds of postoperative adjuvant RT or CCRT; however, these patients still exhibited a worse prognosis than patients with a low LNR. Interestingly, patients with a high LNR who underwent adjuvant RT or CCRT had a better long-term survival than patients who underwent surgery alone. LNR alone not only stratified high-risk patients according to prognosis but also identified patients who may benefit from adjuvant treatment. When determining the best adjuvant treatment strategy for only patients with a high LNR, CCRT was not superior to RT because only a 5% improvement in 5-year DSS was observed. Amazingly, when the LNR and number of positive lymph nodes were combined, patients with an LNR > 0.075 and more than 2 positive lymph nodes and who underwent only adjuvant CCRT exhibited improvements in 5-year DFS. The conclusion was meaningful because the National Comprehensive Cancer Network (NCCN) only describes the presence of ECS and/or positive margins in head and neck cancer as an indication for CCRT. A further prospective clinical trial analysing whether the combination of the LNR and the number of positive lymph nodes can be used to identify whether the benefits of adjuvant CCRT will be helpful for validating this conclusion.

In this study, the clinical T stage coupled with the LNR and ECS were independent prognostic factors for pN + patients with O/OPSCC. The T stage has a role in determining prognosis because all patients were selected based on positive lymph node status. Notably, the LNR status is positively correlated with the T stage, further indicating that a high LNR is a high-risk prognostic parameter.

The incidence of HPV-related oropharynx carcinoma is continuously increasing worldwide, as approximately 60% of US patients with oropharyngeal cancer are HPV-positive. HPV-positive patients are now recognized as a distinct subgroup of patients with oropharyngeal carcinoma and are associated with an improved response to treatment and better prognosis than HPV-negative patients^24^. However, our previous studies have shown that HPV-positive oropharyngeal carcinoma is rare in China (less than 20%)^25, 26^. In this study, tumours located in the oropharynx have a lower LNR than tumours located in the oral cavity. According to the results of the Kaplan-Meier analysis, oropharyngeal cancer has a similar prognosis as oral cancer. The results were not consistent with reports from Europe and the USA, where most cases of oropharyngeal cancers are related to HPV infection and often have a later N stage and better prognosis. The main cause for this difference was that most Chinese patients with oropharyngeal cancer in the study sample were HPV-negative. Therefore, oropharynx and oral squamous cell carcinoma cases in China were pooled in this study, and the results should be more reasonable than the results from Europe and the USA.

This retrospective study has inherent limitations. Data were not available for some important baseline factors, including the number of ECS, the depth of tumour invasion and tumour thickness. Another limitation was that the HPV status was not analysed in the majority of cases of oropharyngeal cancers and the choice of adjuvant therapy did not refer to the HPV status. These limitations will be considered in future studies. In this study, we excluded patients with an LNY of less than 10 to reduce the statistical bias from unqualified cases, as reported by Prabhu *et al*.^10^. Furthermore, patients with pN3 disease were also excluded from this study because of the presence of uncountable matted lymph nodes, as we previously reported^14^. Based on these considerations, the design of our study was rigorous.

Cancer is a complex disease, and its development is driven by inherent oncological molecular features. In the future, the identification of crucial gene signatures will represent a greater advantage in personalized medicine compared with traditional clinicopathological factors^27^. Based on results from our recent studies, several potential biomarkers, such as cyclin D1, enhancer of zeste homologue 2 and glycerol-3-phosphate dehydrogenase 1-like gene, can assist in guiding adjuvant therapy decisions and stratifying high-risk prognostic populations and might have a more accurate efficiency than clinical the TNM stage^18, 25, 28, 29^. The concept of precision medicine based on translational research and novel pathological features is the most promising breakthrough that will further improve the quality of life and long-term survival of patients with head and neck cancer.

In conclusion, an LNR equal to 0.075 is the best cut-off value for predicting 5-year DFS. A high LNR is closely correlated with adverse parameters that markedly hinder favourable prognoses. The LNR and ECS are superior to traditional TNM staging, and the combination of the LNR with the number of positive lymph nodes predicts the benefits of adjuvant CCRT and improves the power of prognostic judgement.

## Patients and Methods

### Patients

This study was conducted in full compliance with ethical principles, including the World Medical Association Declaration of Helsinki (2002 version), and with the approval of the Institutional Review Board of the Beijing Stomatological Hospital of Capital Medical University. Due to the retrospective nature of this study, exemption was granted for obtaining written informed consent from the subjects. The investigators designed and implemented a retrospective cohort study to address the research aim. The study population comprised all patients who were treated in the Department of Oral and Maxillofacial-Head and Neck Oncology, Beijing Stomatological Hospital, Capital Medical University, and were pathologically diagnosed with O/OPSCC between January 2000 and August 2015. For inclusion in the study sample, the patients must fulfil the following criteria: (1) the patients underwent neck dissection (level I-III, level I-IV or level I-V) with pathological lymph node metastasis; (2) the LNY and the number of positive lymph nodes were available; (3) primary tumours were located in the tongue, lower or upper gingiva, buccal mucosa, floor of the mouth, oropharynx and hard palate; and (4) there was no evidence of distant metastasis. Patients were excluded if they had received preoperative chemotherapy/radiotherapy, had unresectable disease, had an LNY less than 10, or has a lack of adequate information to determine the LNY^14^. Patients with clinical N3 disease were also excluded due to the difficulty in distinguishing a largely single metastatic lymph node from uncountable matted lymph nodes, as reported in our recently published article^15^.

### Variables

The predictor variable was the LNR, which was calculated by dividing the number of positive lymph nodes by the number of lymph nodes on the side of the neck characterized by metastatic disease, regardless of whether the patients had undergone bilateral neck dissection. The parameters used to assess the main outcomes were 5-year DFS and 5-year DSS. DFS was calculated as the length of time from diagnosis until the first documented recurrence or death. DSS was calculated as the time from the first operation to the time of death or last follow-up; patients who died from causes other than OSCC were defined as survivors at the time of death. Other variables included demographic (age and sex), anatomical (T stage, growth pattern, and sites), habitual (tobacco and alcohol use) and pathological (LNY, number of positive lymph nodes, pN status, ECS and grade) variables.

### Treatment and neck specimens

All patients were initially treated with surgery. The surgical procedure was selected by the surgeon according to the tumour site and local practice. Standard surgery, including radical tumour resection, neck dissection and the reconstruction of tissue defects (as necessary), was performed. Local excision of the primary site was performed with a minimum margin of 15 mm.

Neck dissection specimens were resected en bloc, and each nodal level was placed in a separate group. The surgical specimens were carefully palpated, and all identified lymph nodes were counted, sectioned at 2- to 3-mm intervals, and embedded in paraffin. Standard haematoxylin and eosin (H&E) staining was performed. All lymph node-related data were derived from original pathology reports. The seventh edition of the TNM staging system for OSCC was used for nodal staging^30^.

Postoperative RT to the neck was advised for all patients. A conventional radiotherapy regimen, which consisted of five 200-cGy fractions per week administered from Monday to Friday, was followed. The total dose for the primary tumour bed and involved neck nodes was >6000 cGy. CCRT with cisplatin (30 mg/m^2^ weekly) was recommended for patients with multiple pathological nodal metastases and/or ECS.

### Data analyses

The cut-off date for all surviving patients was November 2016. Descriptive statistics were summarized as frequencies, percentages, and means ± standard deviations. ROC curves, the AUC, sensitivity, specificity and 95% CIs were calculated to determine which LNR best defined the different risk groups of patients with O/OPSCC. ROC curve analyses were performed using MedCalc software, version 10.4.7.0 for Windows (MedCalc, Ostend, Belgium). All patients were classified into binary subgroups using the best LNR value and the number of positive lymph nodes as the cut-off points, which were directly calculated using MedCalc software.

The Kaplan-Meier method was used to derive estimates of the 5-year DSS. Statistical significance was determined using the log-rank test. A Cox proportional hazards model was used to adjust for the effects of other potential confounders. All tests were two-sided, and *P* values less than 0.05 were considered statistically significant. All statistical analyses, with the exception of the ROC curve analysis, were performed using SPSS software, version 17.0 for Windows (SPSS, Chicago, IL, USA).
